# Decreased Functional Connectivity of Vermis-Ventral Prefrontal Cortex in Bipolar Disorder

**DOI:** 10.3389/fnhum.2021.711688

**Published:** 2021-07-16

**Authors:** Huanhuan Li, Hu Liu, Yanqing Tang, Rongkai Yan, Xiaowei Jiang, Guoguang Fan, Wenge Sun

**Affiliations:** ^1^Department of Radiology, The First Hospital of China Medical University, Shenyang, China; ^2^Department of Psychiatry, The First Hospital of China Medical University, Shenyang, China; ^3^Department of Radiology, The Second Affiliated Hospital of Hainan Medical University, Haikou, China; ^4^Department of Radiology, Johns Hopkins University School of Medicine, Baltimore, MD, United States; ^5^Brain Function Research Section, The First Hospital of China Medical University, Shenyang, China

**Keywords:** bipolar disorder, resting state, functional connectivity, cerebellum, vermis

## Abstract

**Objectives**: To investigate changes in functional connectivity between the vermis and cerebral regions in the resting state among subjects with bipolar disorder (BD).

**Methods**: Thirty participants with BD and 28 healthy controls (HC) underwent the resting state functional magnetic resonance imaging (fMRI). Resting-state functional connectivity (rsFC) of the anterior and posterior vermis was examined. For each participant, rsFC maps of the anterior and posterior vermis were computed and compared across the two groups.

**Results**: rsFC between the whole vermis and ventral prefrontal cortex (VPFC) was significantly lower in the BD groups compared to the HC group, and rsFC between the anterior vermis and the middle cingulate cortex was likewise significantly decreased in the BD group.

**Limitations**: 83.3% of the BD participants were taking medication at the time of the study. Our findings may in part be attributed to treatment differences because we did not examine the effects of medication on rsFC. Further, the mixed BD subtypes in our current study may have confounding effects influencing the results.

**Conclusions**: These rsFC differences of vermis-VPFC between groups may contribute to the BD mood regulation.

## Introduction

Bipolar disorder (BD) is a severe psychiatric illness characterized by recurrent disturbances in sleep, behavior, perception, cognition, and mood regulation (Goodwin and Geddes, 2007). The cerebellum has long been regarded as a brain structure involved in motor systems (anterior lobe and lobule VI), there is growing contemporary evidence that it influences cognition (posterior lobe) and mood regulation (the vermis; Schutter and Van Honk, 2005; Schmahmann, 2019). The cerebellum’s involvement in mood regulation is consistent with earlier clinical studies that suggested the cerebellum functioned as an emotional pacemaker (Heath, 1977; Heath et al., 1979), as well as contemporary evidence that implicates the cerebellar vermis and fastigial nucleus as the limbic cerebellum (Schmahmann, 2001, 2004). The fastigial nucleus, one of the deep cerebellar nuclei, mediates the connection between the vermis and the cerebellar inferior peduncle and connects to the reticular formation and the limbic system through the inferior peduncle (Schmahmann, 2004). The connections between the vermis and both the reticular and limbic system imply that the vermis plays an important role in the regulation of affect (Stoodley and Schmahmann, 2009; Moulton et al., 2011). Some studies found that multi-episode BD patients have smaller vermal V2 and V3 areas *via* structural magnetic resonance imaging (MRI) compared to first-episode patients (DelBello et al., 1999; Mills et al., 2005). These data suggested that the vermis might therefore be subject to atrophy during BD spells. Moreover, mood disorders such as BD have been linked to impairments in anterior limbic brain structures, wherein the cerebellum may modulate mood (Strakowski et al., 2002).

Recent studies of spontaneous resting-state functional connectivity (rsFC) have focused on the BD brain network abnormalities such as abnormal rsFC in the frontotemporal system (Chepenik et al., 2010; Dickstein et al., 2010) and corticolimbic system (Anand et al., 2009). rsFC between the cerebellum and the whole brain can also be defined as the temporal dependency of their neural activation patterns by their coherence in spontaneous fluctuations in resting-state functional MRI (fMRI) signals (Buckner and Vincent, 2007). One recent MRI study found that the cerebellum and basal ganglia are closely correlated with mood states in BD, representing the altered metabolic activity of BD patients’ cerebellum (Johnson et al., 2018). Another resting-state fMRI study also found altered cerebellum-brain region connectivity in unmedicated BD (Chen et al., 2019).

In this study, we utilized a region-of-interest (ROI) based approach to examine rsFC in individuals with BD and healthy control (HC) participants. We selected the vermis as ROI and hypothesized that the BD group would show altered rsFC between vermis and cerebral regions which are involved in mood regulation compared to the HC group.

## Materials and Methods

### Subjects

All BD participants were diagnosed using the Structured Clinical Interview for DSM-IV (Bell, 1994) and fulfilled DSM-IV criteria for BD in this study. Using DSM-IV criteria, psychologists of our working group recruited all BD patients from the outpatient center of the First Hospital of China Medical University and Mental Health Center of Shenyang between June 2010 and August 2018. Enrolled patients were consistently aged 18–50 years right-handed, and exhibited neither neurological illness nor head trauma involving loss of consciousness exceeding 5 min, nor any major physical disorder or contraindication for fMRI scanning. Psychologists systematically evaluated the presence or absence of Axis I Disorder for the recruited patients and assessed patients’ mood state at scanning according to the DSM-IV Structured Clinical Interview. Psychological examinations of all HCs recruited from the local community were normal, these examinations confirmed no personal histories of mental illness, mood, psychotic, anxiety, or substance misuse disorders in their first-degree family members. Thirty BD patients and 28 HCs were ultimately included in the study population (matched by age and gender, *p* > 0.05). Symptoms were assessed using the Hamilton Depression Rating Scale (HDRS) and the Young Mania Rating Scale (YMRS). Twenty-five (83.3%) of the BD participants were taking medication at the time of scanning. Some of the participants in this study also participated in our previous study (Xu et al., 2014). Their behavioral assessment was made by XJ. All participants were approved by the ethics committee of the first hospital of China Medical University and provided a signed, written informed consent.

At the time of scanning, five (16.7%) participants with BD met DSM-IV criteria for a depressive episode and six (20.0%) for a manic/mixed or hypomanic episode, whereas the remaining 19 (63.3%) were euthymic. Detailed demographic and clinical characteristics of the participants are presented in Table 1.

**Table 1:** Demographic and clinical data of subjects.

	Healthy	Bipolar disorder	*P*
*N*	28	30	NA
Age (years, mean ± SD)	31.38 ± 8.08	30.51 ± 8.79	0.836
Sex (male: female)	13:15	18:12	0.300
HDRS (mean ± SD)	0.40 ± 0.77	9.71 ± 10.12	<0.001
YMRS (mean ± SD)	0.06 ± 0.35	6.43 ± 9.33	<0.001
Medication(yes/no)	NA	25/5	NA
Typical antipsychotics *(N)*	NA	16	NA
Anticonvulsant *(N)*	NA	14	NA
Lithium salts *(N)*	NA	5	NA
Antidepressants *(N)*	NA	11	NA

*SD, standard deviation; HDRS, Hamilton Depression Rating Scale; YMRS, Young Mania Rating Scale; NA, not applicable; N, number*.

### MRI Scanning and Image Preprocessing

All fMRI scans were performed using a 3.0-T GE Signa System (GE Signa, Milwaukee, Wisconsin, USA) in the Department of Radiology, the First Hospital of China Medical University. The clinician asked the patients to remove any metal jewelry or accessories that might interfere with the machine and briefly introduced the procedure of MRI scanning to reduce the anxiety of patients. Foam pads were provided to reduce head motion and scanner noise when patients were lying down. Technician set the parameter of a 3D-SPGR sequence to acquire three-dimensional T1-weighted images in a sagittal orientation with the repetition time (TR) = 7.1 ms, echo time (TE) = 3.2 ms, field of view (FOV) = 24 cm×24 cm, flip angle = 15°, matrix = 256 × 256, slice thickness = 1.8 mm, no gap. The fMRI scanning was performed in darkness, and an observer stood to one side to ensure the patients kept their eyes closed, relaxing, and moving as little as possible. The slices of functional images were positioned approximately along the AC-PC line using a gradient echo-planar imaging (EPI): TR = 2,000 ms, TE = 30 ms, FOV = 24 cm × 24 cm, flip angle = 90°, matrix = 64 × 64, slice thickness = 3 mm, no gap, slices = 35. For each participant, the fMRI scanning lasted 7 min. Image preprocessing was carried out using SPM8^1^ and DPABI (Yan et al., 2016). Preprocessing consisted of slice-time correction, motion correction, spatial normalization, and spatial smoothing full width at half maximum (FWHM = 6 mm). Movement parameters were extracted out by SPM8 for each participant, which can exclude the data sets with more than 2 mm maximum translation along the x, y, or z axes, allowing 2° of maximum rotation about three axes among each image. Further preprocessing consisted of removing linear drift through linear regression and temporal band-pass filtering (0.01–0.08 Hz) to reduce the effects of low-frequency drifts and physiological high-frequency noise.

### Definition of ROIs

**Figure 1:**
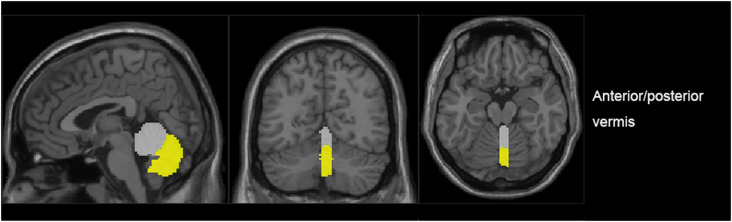
The generated anterior (gray) and posterior (yellow) regions of interest (ROIs) in a representative subject.

The vermis was divided into anterior vermis (vermis I-V) and posterior vermis (vermis VI-IX) by AAL (Anatomical automatic labelling; Pfefferbaum et al., 2011; Figure 1). For each ROI, the blood oxygen level dependence (BOLD) time series of the voxels within the ROI were averaged to generate the reference time series.

A whole-brain mask was created by taking the intersections of the normalized T1-weighted high-resolution images of all participants, which were stripped using the software BrainSuite2^2^.

### FC Analysis

A regression generalized linear model (GLM) was created for each participant, including a time series regressor for one of the two vermal subregions, and applied to each of eight nuisance covariates (white matter, cerebrospinal fluid, and six motion parameters). Correlation analysis was performed in a voxel-wise manner between the seed ROIs and the whole brain using DPABI. The correlation coefficients were then transformed to *z*-values using the Fisher *r*-to-*z* transformation for more conforming to Gaussian distribution. A one-sample *t*-test model was used to delineate the functional connectivity of each vermis ROI in the first-level analysis. Direct comparisons were conducted to identify differences in functional connectivity between BD vs. HC in the second-level random-effects analysis.

### Statistical Analyses

Statistical significance was determined by a corrected *P* < 0.05 that combined individual voxel *p*_(uncorrected)_ < 0.01 with GRF (Gaussian random field) correction for cluster-level inference of *p* < 0.05 (Bousse et al., 2012). Additional exploratory analyses (ANCOVA) were performed for effects of medications (overall presence or absence of medication) on the regions that showed significant differences between the HC and BD groups. Finally, significant correlations between HDRS, YMRS in the BD group, and the transformed *z*-scores showing significant group differences were performed using exploratory correlation analyses to identify the relationship between the symptom severity and the strength of connectivity. A two-tailed *p* level of 0.05 was used as the criterion of statistical significance.

## Results

**Figure 2:**
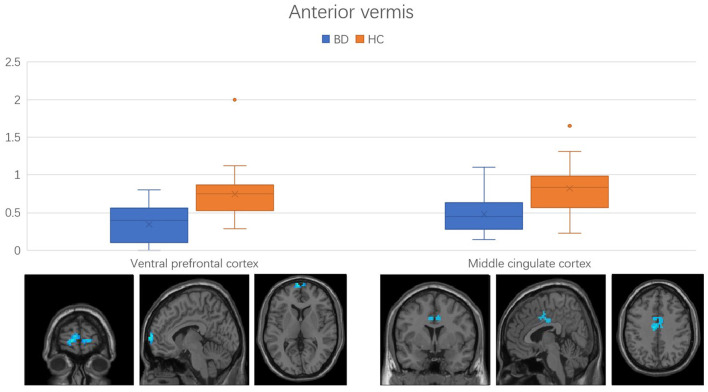
Functional connectivity between anterior vermis and ventral prefrontal cortex, middle cingulate cortex in the comparison between bipolar disorder (BD) and healthy controls (HC) groups. Error bars represent the standard deviation of Z values at the peak voxel. BD, bipolar disorder; HC, healthy control.

**Figure 3:**
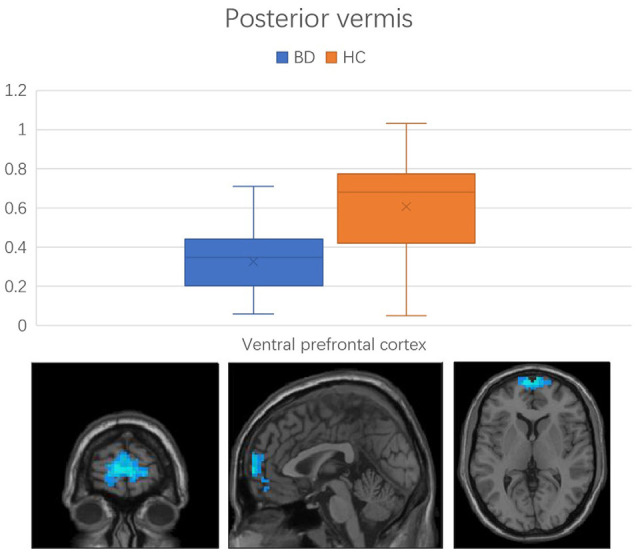
Functional connectivity between the posterior vermis and ventral prefrontal cortex in the comparison between BD and HC groups. Error bars represent the standard deviation of *Z* values at the peak voxel. BD, bipolar disorder; HC, healthy control.

Regions with changed vermal connectivity between the BD and HC groups are shown in Table 2. Compared to the HC group, significant differences in rsFC were observed between the anterior vermis and brain regions that included ventral prefrontal gyrus (VPFC; BA 10) and middle cingulate cortex (BA 24; Figure 2), while the posterior vermis showed significant differences in rsFC with VPFC (BA 10) in the BD group (Figure 3). In addition, there were no significant effects of medication on FC values in the regions that differed between the HC and BD groups (ANCOVA test, *p* > 0.05). Finally, correlation analysis was performed between the connectivity coefficient within clusters showing significant group differences and behavioral measures as assessed by HDRS, YMRS in the BD group. Analyses of correlations did not show any significant effects between functional connectivity and clinical scores (Table 3).

**Table 2:** Detailed information for clusters showing group connectivity differences in BD at the given threshold (cluster size > 297 mm^3^, and *P* < 0.00014).

Voxels	PV_X	PV_Y	PV_Z	H	Brain regions (AAL atlas)	BA	*T*
Anterior vermis				
104	−6	68	7	L	Ventral prefrontal cortex (Frontal_superior_medial)	10	−5.030
190	7	−16	32	R	Middle cingulate cortex (Cingulum_middle)	23	−4.160
Posterior vermis					
201	−4	69	5	L	Ventral prefrontal cortex (Frontal_superior_medial)	10	−4.430

*PV: peak voxel. *X, Y, Z*: coordinates in the Montreal Neurological Institute space. BA: Brodmann area. *T*: *T* values from a *t*-test of the peak voxel (showing greatest statistical difference within a cluster)*.

**Table 3:** The correlation between the strength of these changed connectivity regions and the clinical scores in BD group.

Brain regions	Clinical scores			
	HDRS	*P*	YMRS	*P*
Changed connectivity with the anterior vermis		
Ventral prefrontal cortex	−0.307	0.332	−0.434	0.213
Middle cingulate cortex	−0.532	0.143	0.125	0.544
Changed connectivity with the posterior vermis		
Ventral prefrontal cortex	−0.631	0.095	0.154	0.510

*The numbers in the table are Pearson’s correlation coefficients*.

## Discussion

The current study examined vermal connectivity in BD patients. We discovered that two cerebral regions (VPFC and middle cingulate cortex) showed decreasing connectivity with the vermis. Previous studies show that these two brain regions exhibited changed neural activity or disturbed connectivity with other cerebral regions. We initially found the connectivity pattern between vermis and these two cerebral regions was similarly disturbed in BD patients.

Previous studies proposed that the vermis can be considered as the “limbic cerebellum,” based on its regional connections with limbic structures (Schmahmann, 2001, 2004). Patients with the cerebellar cognitive affective syndrome can show emotional lability, inappropriate laughing or crying, and changes in affection, suggesting that these cerebellar-limbic connections are involved in the modulation of emotional processing (Levisohn et al., 2000). What’s more, malformations of the posterior vermis have been confirmed to be associated with emotional symptoms (Tavano et al., 2007). These studies implicated the cerebellar vermis, especially the posterior vermis play important roles in mood regulation. Interestingly, the VPFC has now also been shown to play an important role in emotion processes (Kringelbach, 2005). Many fMRI studies have found abnormal activation of the VPFC in BD during tasks (Blumberg et al., 2003; Elliott et al., 2004; Lawrence et al., 2004; Strakowski et al., 2004; Malhi et al., 2005). Abnormal VPFC neural activity and disturbed VPFC-amygdala rsFC were also observed by resting-state studies (Liu et al., 2014; Xu et al., 2014). Trait abnormalities of VPFC in BD are further supported by postmortem histopathological findings such as decreased glial density and reductions in the density of both neurons and glia (Ongur et al., 1998; Rajkowska, 2000, 2002). In our study, the entire vermis showed changed rsFC patterns with the VPFC, establishing that the decreased rsFC of vermis-VPFC plays an important role in the regulation of mood linked to the core psychopathology of BD.

Another changed connectivity region of the anterior vermis, which belongs to the anterior lobe of the cerebellum, is the middle cingulate cortex (BA 24). The function of the anterior cerebellar lobe is mainly associated with motor control (Stoodley and Schmahmann, 2009). The middle cingulate cortex area is the midsection of the cingulate gyrus in its anterior-posterior axis and appears to be involved in both motor control and cognitive tasks such as response selection, error detection, competition monitoring, and working memory (Torta and Cauda, 2011). Previous studies have consistently reported aberrant motor control presentation in BD (Manschreck et al., 2004; Krebs et al., 2010; Deveney et al., 2012; Weathers et al., 2012). Our findings combined with previous studies suggest that the anterior vermis may be involved in the motor control of BD patients, which should be further validated by future studies.

There are several limitations to this study. First, 83.3% of the BD participants were taking medication at the time of the study. Although we did not find significant effects of medication on FC values in this study, our findings may in part be attributed to treatment differences. Second, confounding effects may influence the result of mixed BD subtypes in our current study; future studies that compare subtypes in BD would likely contribute to our understanding of the underlying mechanisms of BD. Thirdly, the sample size is modest. Finally, correlation analyses did not reveal significant relationships between rsFC and symptom measures in BD. In this study, only the HDRS and YMRS symptom measurements were assessed in the BD group. Future studies should include more comprehensive symptom measurements to enhance our understanding of the relationship between symptom severity and functional connectivity as well as state vs. trait-related abnormalities in BD. Because the majority of BD participants in this study were in remitted states, our findings more likely reflect trait-related differences between BD and HC.

## Conclusion

In summary, BD patients showed decreased rsFC of vermis and VPFC as compared to the HC group. This resting-state fMRI study suggests that the abnormal rsFC of vermis-VPFC may contribute to mood regulation in BD patients. Further work focusing on this field may contribute to our understanding of BD neuropathphysiology.

## Data Availability Statement

The raw data supporting the conclusions of this article will be made available by the authors, without undue reservation.

## Ethics Statement

The studies involving human participants were reviewed and approved by The First Hospital of China Medical University. The patients/participants provided their written informed consent to participate in this study.

## Author Contributions

Conception and design: GF and WS. Development of methodology: HLiu. Data acquisition, analysis, and interpretation: YT and XJ. Writing, review, and/or revision of the manuscript: HLiu, HLi, and RY. Study supervision: YT, GF, and WS. All authors contributed to the article and approved the submitted version.

## Conflict of Interest

The authors declare that the research was conducted in the absence of any commercial or financial relationships that could be construed as a potential conflict of interest.

## Footnotes

^1^http://www.fil.ion.ucl.ac.uk/spm

^2^http://brainsuite.org

## Abbreviations

BD: Bipolar disorder
MRI: Magnetic resonance imaging
rsFC: Resting-state functional connectivity
fMRI: functional MRI
ROI: Region of interest
HC: Healthy control
HDRS: Hamilton Depression Rating Scale
YMRS: Young Mania Rating Scale
BOLD: Blood oxygen level dependence
GLM: Generalized linear model
FWHM: Full width at half maximum
VPFC: Ventral prefrontal gyrus.
